# Video-dominant emotion recognition for portable EEG-based devices

**DOI:** 10.1038/s41598-026-39315-8

**Published:** 2026-02-09

**Authors:** Xinyi Wen, Wei Xu, Lei Tian, Cuijuan Guo, Jinjun Bai

**Affiliations:** 1 Tianjin Key Laboratory of Optoelectronic Detection Technology and System, School of Electronics and Information Engineering, Tiangong University, Tianjin, 300387 China; 2 School of Life Sciences, Tiangong University, Tianjin, 300387 China

**Keywords:** Engineering, Mathematics and computing, Neuroscience

## Abstract

Electroencephalography (EEG) signals offer a promising avenue for detecting emotional responses during video viewing, enabling the automated recognition of video-induced emotions and providing an objective assessment approach. However, current approaches face two main limitations. First, emotion labels often rely on subjective self-reports that introduce personal bias. Second, most systems require high-density electrode arrays that are costly and impractical for portable applications. To address these challenges, this study explores video emotion recognition using a lightweight EEG setup. We introduce three complementary strategies: (i) a dynamic hierarchical label calibration approach that reduces labeling subjectivity through consistency modeling and boundary refinement; (ii) a multi-dimensional energy ratio analysis that compresses channel requirements while preserving discriminative information; and (iii) a saliency-guided feature selection method to improve generalization capability. By reducing 65% of the channels from the original dataset, our approach achieves 45% accuracy in four-class dominant video emotion prediction using only 11 channels, while maintaining meaningful discriminative performance under cross-subject conditions. Beyond technical advancements, these results demonstrate the potential of EEG-based systems to capture collective emotional responses to video content. This capability supports practical applications in audience sentiment analysis, media content evaluation, and emotion-aware recommendation systems.

## Introduction

EEG is a non-invasive technique for recording brain electrical activity and has been widely applied in neuroscience, clinical diagnosis, and brain–computer interface research. In recent years, the use of EEG to analyze subjective emotional responses during video viewing has attracted increasing attention in the field of affective computing^1^. Video stimuli play a central role in applications such as advertising, film and television recommendation, public opinion analysis, and psychological intervention^2,3^, where the accurate identification of dominant audience emotions is critical to content effectiveness and dissemination.

Recent high-impact studies have advanced EEG-based emotion recognition on the DEAP dataset by exploiting fine-grained temporal representations and deep sequence modeling techniques^4–6^. These methods, primarily developed under subject-dependent settings, aim to maximize classification accuracy at the trial or short-window level and represent the current state of the art in data-driven emotion decoding. Despite this progress, several challenges continue to limit the practical deployment of EEG-based video emotion recognition systems. First, emotion labels are typically derived from subjective self-assessments, which exhibit substantial inter-subject variability and limited cross-subject generalizability^7^. Most existing approaches rely on valence–arousal representations^8,9^, or extend them to four-dimensional rating schemes incorporating dominance and liking^10,11^, yet these formulations remain insufficient to fully capture group-level emotional consistency. Second, many state-of-the-art models depend on high-dimensional feature sets and complex network architectures^13,14^, resulting in considerable computational overhead and reduced suitability for real-time applications. Third, conventional high-density EEG systems are costly, bulky, and operationally demanding^15,16^, which constrains their use in portable or large-scale deployment scenarios.

To address these limitations, this study focuses on video-induced emotion recognition using portable EEG devices. Our main contributions are threefold. First, we propose a dynamic hierarchical label calibration strategy that mitigates label subjectivity by adaptively refining decision boundaries for ambiguous samples, thereby improving label consistency. Second, we introduce a multi-dimensional energy ratio analysis framework to optimize EEG channel utilization, reducing hardware complexity by more than 65% while maintaining competitive classification performance. Third, a saliency-guided feature selection strategy is developed to extract robust spatiotemporal–frequency features for emotion recognition. Together, these components form a practical framework for four-class video emotion recognition using portable EEG systems, with potential applications in audience sentiment analysis, personalized content recommendation, and emotion-aware video screening.

## Related work

Table 1 summarizes representative studies based on the Database for Emotion Analysis using Physiological Signals (DEAP)^17^, comparing dataset configurations, methodological choices, EEG channel usage, and key findings. The majority of prior work employs the full 32-channel EEG setup and focuses on binary valence or arousal classification under subject-dependent evaluation protocols, where relatively high performance can be achieved in controlled experimental settings. In contrast, existing studies consistently report substantial performance degradation when channel reduction and cross-subject evaluation are jointly considered, particularly for multi-class emotion recognition tasks.

In this context, the present study targets a more realistic and practically relevant scenario by investigating four-class valence–arousal quadrant recognition using only 11 channels from a portable EEG configuration under cross-subject evaluation. Rather than pursuing maximal classification accuracy, our results demonstrate that informative and discriminative emotional patterns can still be extracted under reduced-channel constraints. This establishes a practical baseline for portable EEG-based emotion recognition in real-world applications. By integrating dynamic hierarchical label calibration, multi-dimensional energy ratio analysis, and saliency-guided feature selection, the proposed framework effectively mitigates inter-subject variability, optimizes channel utilization, and enhances the robustness of spatiotemporal–frequency feature representations. Collectively, these design choices support the feasibility of video-dominant emotion recognition using portable EEG devices and highlight their potential utility in audience sentiment analysis, content recommendation, and emotion-aware video screening, even under challenging cross-subject and low-channel conditions.

**Table 1:** Representative DEAP-based studies with key findings.

Study	Features / methodology	#Ch	Task	Key findings / notes
Lin et al. ^18^	Transfer learning with spectral or time features	32	Binary	Reported moderate accuracy around 50% under cross-subject settings, highlighting the difficulty of generalization.
Apicella et al. ^19^	Dry-EEG single or low-channel model	1–8	Binary	Showed feasibility of using few electrodes; accuracy around 60% but limited to subject-dependent protocols.
Galvão et al. ^20^	Handcrafted features Regression models	32	4-class	Achieved high accuracy greater than 80% in subject-dependent evaluation using the full-channel setup.
Moctezuma et al. ^21^	CNN + NSGA-II channel selection	1–32	4-class	Ablation with 1–15 channels showed sharp degradation; authors note that cross-subject + reduced-channel setups yield much lower performance around 40%.

### Construction of emotion labels

Emotion labels constructed under a uniform evaluation mechanism often suffer from strong subjectivity, primarily due to substantial differences in emotional expression across individuals with diverse ages, cultural backgrounds, and ethnicities. Such label bias can significantly degrade model performance and generalization capability, particularly in cross-subject emotion recognition tasks. To alleviate labeling inconsistency, various label correction and consistency enhancement strategies have been proposed in prior studies. For instance, Koelstra et al. ^22^ combined subjective ratings with physiological signals in the DEAP dataset to assist emotion annotation, while Yin et al. ^23^ incorporated multimodal information and integrated learning frameworks to improve recognition accuracy. In addition, neuromorphic-inspired studies have explored emotion-related learning mechanisms at the circuit level^24,25^. Although these approaches improve label quality to a certain extent, they typically involve high computational cost and complex post-processing procedures, and remain limited in addressing individual variability and cross-subject label consistency. In contrast, the dynamic calibration strategy proposed in this work provides an efficient mechanism to refine constructed emotion labels by improving their alignment with the original subjective sentiment references, thereby enhancing label consistency without introducing excessive computational overhead.

### Analysis of EEG signals

To characterize emotion-related neural activity, EEG signals are commonly decomposed into five standard frequency bands, each corresponding to distinct functional states of the brain^26,27^. As a highly irregular multivariate time series^28,29^, EEG presents substantial challenges for effective feature representation. In recent years, self-supervised learning has emerged as a promising direction for enhancing EEG feature extraction and representation learning^30,31^. Related studies have also investigated finite-time stability properties in EEG-based systems^32^. Meanwhile, DaŞdemir et al. ^33,34^ explored EEG-based emotion classification in immersive virtual reality and audiovisual stimulation environments. Most existing EEG emotion recognition methods rely on features extracted from the time domain, frequency domain, or time–frequency domain, each with distinct advantages and limitations. Wang et al. ^35^ demonstrated that power spectral features outperform conventional statistical descriptors, particularly in the $\alpha$ and $\beta$ bands, while Momennezhad et al. ^36^ employed wavelet energy ratios to effectively discriminate among multiple emotional states. However, several limitations persist in existing approaches: interactions between different frequency bands are often overlooked; systematic feature alignment and dimensionality reduction mechanisms are insufficiently explored; and cross-channel energy differences are not adequately modeled in high-dimensional EEG data. To address these issues, this study introduces a multi-dimensional energy ratio analysis strategy that explicitly captures inter-band and inter-channel energy relationships, thereby enhancing feature discriminability and improving model robustness.

### Feature selection strategy

Most existing EEG-based emotion recognition studies rely on 32-channel or higher-density acquisition systems. While such configurations can improve recognition accuracy, they are expensive, bulky, and operationally complex, which limits their suitability for practical deployment in wearable, home-based, or mobile scenarios. Moreover, high-channel EEG systems often exhibit reduced generalization performance under low-cost and low-power constraints^37^. Consequently, effective channel compression while preserving recognition performance has become a critical research direction for practical emotion recognition systems. Previous studies have explored channel selection and model compression strategies to address this challenge. For example, Wu et al. ^38^ demonstrated emotion recognition using only two prefrontal channels, though their approach focused solely on valence and did not capture other emotional dimensions. Zhu et al. ^39^ employed simplified graph convolutional networks to enable channel recalibration and adaptive graph optimization; however, the resulting model complexity increases deployment cost and reduces interpretability. These findings indicate that appropriate channel selection and region-focused strategies can effectively balance recognition performance and device portability. Building upon saliency-guided learning mechanisms^40,41^ and hierarchical reinforcement learning approaches^42,43^, this work integrates these methodologies into a unified framework tailored for portable EEG systems. Accordingly, a saliency-guided feature selection strategy is developed that jointly considers signal discriminability and hardware implementation cost.

## Methodology

**Fig. 1:**
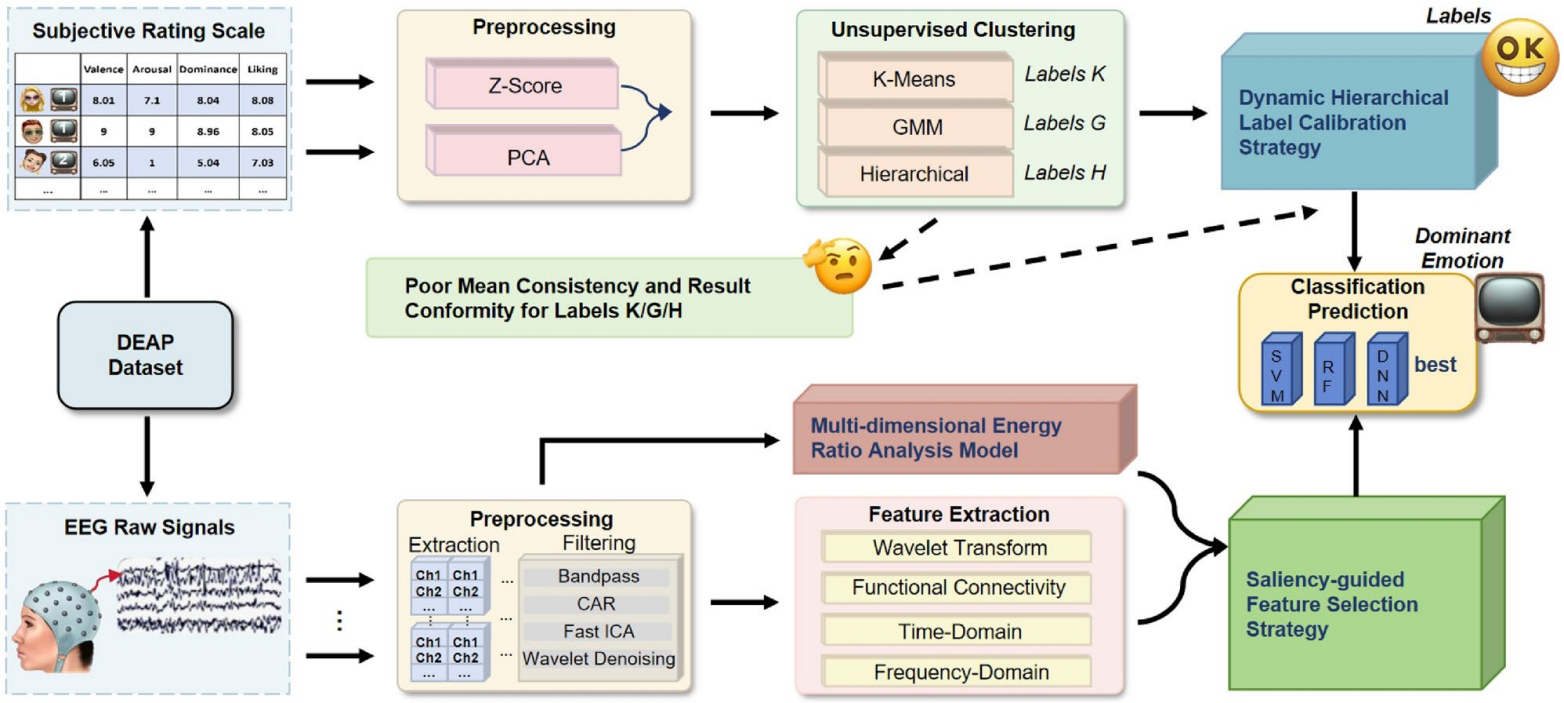
Overall framework of proposed method.

Figure 1 illustrates the flow of a video-dominant emotion recognition study for a portable EEG device. The process starts with the DEAP dataset, which adopts a controlled video-based emotion elicitation paradigm, in which participants watch a set of standardized music video clips and subsequently provide subjective ratings along the Valence, Arousal, Dominance, and Liking (VADL) dimensions.For each participant, multi-channel EEG signals are recorded continuously during video presentation, together with corresponding self-assessment scores. In this work, we follow the original experimental protocol and rating criteria provided by DEAP, and further reorganize the data to support video-level dominant emotion modeling. Specifically, EEG recordings originally structured by subject are re-grouped according to emotion categories derived from video-level ratings. All EEG segments associated with the same emotional video condition are integrated across subjects, enabling subsequent consistency analysis and group-level modeling of video-induced emotional tendencies.The original DEAP labels and rating values are preserved, and no additional subjective annotation is introduced.

The raw EEG signals are extracted from each channel and preprocessed by band-pass filtering and common average reference, while the subjective rating scales are subjected to Z-score normalization and principal component analysis. The pre-processed EEG signals were extracted by wavelet transform and other methods, and then analyzed by multidimensional energy ratio analysis, and then channel downscaling and feature selection were performed by a significance-guided module. Afterwards, different labels are obtained by unsupervised clustering methods such as K-means clustering, Gaussian mixture model, hierarchical clustering, etc., but these labels suffer from poor average consistency and result consistency. The labels are corrected to determine the dominant sentiment using dynamic hierarchical label correction strategy, and finally the best model is determined by classification prediction using models such as Support Vector Machines (SVM), Random Forests (RF), and Deep Neural Networks (DNN). Besides, to ensure high recognition accuracy while enhancing system portability and deployment efficiency, this study selects 11 key channels from the original 32-channel EEG configuration in the DEAP dataset for focused analysis. The selected electrodes and their specific rationales are shown in Table 2.

**Table 2:** Key electrodes selection and their functional roles in emotion recognition.

Brain region	Selected electrodes	Primary functions in emotion recognition
Prefrontal cortex	FP1, FP2, F3, F4	Emotional regulation, valence assessment, executive control, and affective decision-making processes
Central cortex	C3, C4	Discrimination of high and low arousal states, sensorimotor integration, and detection of physiological activation
Temporal cortex	T7, T8	Auditory emotional processing, emotional memory encoding, and interactions with amygdala regions
Parietal cortex	Pz	Attentional modulation during emotional stimuli processing and suppression of ocular artifacts
Occipital cortex	O1, O2	Visual processing of emotional stimuli and distinction of emotionally salient visual content

### Dynamic hierarchical label calibration strategy

In EEG-based emotion recognition research, accurately defining the dominant emotion label for each video stimulus is essential to capture its group-level emotional effect. Unlike traditional short-term modeling focused on immediate responses to isolated stimuli, this work emphasizes the overall affective trend elicited by the entire video segment. We propose a dynamic hierarchical label refinement strategy that constructs dominant emotion labels across multiple subjects based on a four-dimensional emotional rating system. This method integrates multidimensional feature compression, unsupervised clustering, and cross-subject consistency analysis to systematically transform individual ratings into group-level dominant emotion representations. Initially, the four-dimensional subjective ratings (Valence, Arousal, Dominance and Like) for each subject are standardized via z-score normalization to eliminate individual differences in rating scales. Principal Component Analysis (PCA) is then applied to reduce dimensionality while preserving the majority of emotional variance. By retaining components explaining 95% of the cumulative variance, an average of three principal components per video are preserved, enhancing clustering robustness and interpretability. Next, unsupervised clustering models—including K-means, Gaussian Mixture Model (GMM), and Hierarchical Clustering—are employed on the reduced feature space. The clustering quality is evaluated comprehensively using silhouette scores and cross-subject consistency metrics. The GMM model is configured to identify four clusters corresponding to the classic two-dimensional Valence-Arousal emotion quadrants. To improve stability, GMM employs 15 random initializations, a full covariance matrix structure, and a regularization factor to prevent singularities. K-means uses multiple random initializations for robustness, while hierarchical clustering adopts Ward’s linkage criterion to ensure compact clusters.

At the group level, each video’s dominant emotion label is determined by the mode of cluster assignments across all subjects, with the label consistency defined by the proportion of subjects sharing this label. Additionally, individual conformity to group labels is assessed via conformity rates, enabling identification of potential labeling outliers. To further improve alignment with original emotion references, a fine-grained adjustment mechanism based on the Dominance and Like dimensions is introduced. After independent z-score normalization of the original ratings, the VA space-based four-class labels are refined by introducing a margin of 0.1 to adjust borderline samples based on their D and L scores. This adjustment addresses classification ambiguities and enhances the psychological interpretability of the labels. Finally, three output modes for dominant emotion labels are provided to support various downstream tasks: majority voting yielding a single definitive label per video, confidence filtering that excludes low-consistency videos for high-confidence analyses, and probabilistic distribution outputs that capture label uncertainty for generative or regression models. This flexible design balances label robustness and adaptability, improving usability compared to rigid single-label assignment methods.

### Multi-dimensional energy ratio analysis model

Many existing studies have used single-channel EEG signals for emotion recognition, which is limited by its difficulty in fully reflecting the spatial synergy patterns of brain activity^44^. Compared to single-channel EEG signals, which only reflect localized brain activity, multichannel analysis captures inter-regional coordination, thereby revealing spatiotemporal coupling patterns of neural activity. The selected 11 channels strike a balance between maintaining emotion-relevant brain region coverage and reducing the hardware complexity—effectively shrinking the original sensor layout by approximately two-thirds while preserving classification performance. The EEG signal preprocessing pipeline begins with a 4th-order Butterworth band-pass filter (1–45 Hz) to remove baseline drift and high-frequency noise. Spatial filtering is then performed using the Common Average Reference (CAR) method, calculated as equal (1):

1 $$\begin{aligned} X_i^{\text {CAR}}(t) = X_i(t) - \frac{1}{N} \sum _{j=1}^{N} X_j(t), \end{aligned}$$

where $X_i(t)$ represents the EEG signal recorded from the $i$-th electrode at time $t$, and $N$ denotes the total number of electrodes. To eliminate ocular and muscular artifacts, Fast Independent Component Analysis (ICA) is applied, and the top four components with kurtosis values exceeding 5 are automatically identified and removed. Finally, the signal is decomposed using a 4-level discrete wavelet transform with the wavelet basis, and denoising is performed using Stein’s Unbiased Risk Estimate (SURE) with soft thresholding. EEG segments corresponding to 60-second video clips are extracted from the DEAP dataset. Based on previous statistical analysis, only signals from subjects whose dominant emotion ratings are above the average are retained and categorized into four emotion classes. For each emotion category, the EEG signals across subjects and videos are aggregated by computing the point-wise median, which reduces the influence of outliers and yields four representative feature sets. Before formal feature extraction, a Power Spectral Density (PSD) analysis is conducted to visualize the energy distribution across channels and frequency bands, thereby guiding subsequent feature selection strategies.

### Saliency-guided feature selection strategy

Although studies have attempted to utilize single-modal features for EEG emotion recognition, there are still limitations in capturing the multidimensional dynamics of the signal. In order to improve the model’s ability to express time-frequency-space multi-features of EEG signals, a multi-modal feature extraction framework was adopted, integrating wavelet energy features, functional connectivity features, time-domain statistical features, and dynamic power spectral features. First, wavelet-based relative energy was computed in standard EEG frequency bands after continuous wavelet transform through Eq. (2):

2 $$\begin{aligned} E_{\text {band}} = \frac{\sum \limits _{f \in \Delta f} \sum \limits _{t=1}^{T} |W(f, t)|^2}{\sum \limits _{f=1}^{60\,\text {Hz}} \sum \limits _{t=1}^{T} |W(f, t)|^2} \times 100\%, \end{aligned}$$

where $W(f, t)$ denotes the wavelet coefficient at frequency $f$ and time $t$, $\Delta f$ represents the target frequency band, and $T$ is the total number of time points. To capture spatial functional coupling, the magnitude-squared coherence was computed between pairs of emotion-related electrodes across different frequency bands as Equation (3):

3 $$\begin{aligned} \text {MSCoh}(f) = \frac{|\mathscr {P}_{XY}(f)|^2}{\mathscr {P}_{XX}(f)\; \mathscr {P}_{YY}(f)}, \end{aligned}$$

where $\mathscr {P}_{XY}(f)$ is the cross-power spectral density between signals $X$ and $Y$, and $\mathscr {P}_{XX}(f)$, $\mathscr {P}_{YY}(f)$ are the auto-power spectral densities of $X$ and $Y$, respectively. From band-pass filtered signals in emotion-relevant channels, statistical features including the median and peak amplitudes were extracted, with a focus on the $\alpha$ and $\beta$ bands. Additionally, to complement the time-domain characterization, a dynamic PSD analysis was conducted using a sliding-window Welch method. Extracted features included the average and maximum power within each frequency band. The final feature vector was constructed by concatenating all four types of features, forming a comprehensive and robust representation of the EEG’s spatiotemporal and spectral information. This multimodal fusion strategy effectively balances local and global characteristics, integrates adaptive artifact removal, and leverages large-scale parallel computing architectures, thus enhancing both the representational richness and classification robustness of the extracted EEG features.

### Classification modeling and prediction

Despite encouraging results reported in prior studies on intra-individual emotion recognition, cross-subject modeling remains subject to a substantial performance bottleneck, primarily due to the pronounced heterogeneity of EEG patterns across individuals^46^. To improve the discriminative capability of EEG features under cross-subject conditions, we developed a comprehensive feature engineering and classification framework, which was systematically evaluated using a five-fold subject-independent cross-validation protocol. Given the considerable inter-subject variability in EEG signal amplitude and feature distributions, feature normalization was performed independently for each subject using z-score standardization. Specifically, features were normalized based on the mean and standard deviation computed from each subject’s own data, resulting in zero-mean, unit-variance representations. This subject-wise normalization strategy effectively reduces the influence of individual differences and facilitates the learning of feature representations with improved cross-subject generalization.

To further capture temporal dynamics and latent variations across feature dimensions, a first-order difference enhancement strategy was introduced. Concretely, first-order differences were computed along the column dimension of the feature matrix (excluding the last column) and concatenated with the original features to form an augmented representation. This operation highlights emotion-related temporal trends in neural responses and enhances the model’s sensitivity to transitions between emotional states. As the augmented feature set substantially increases dimensionality, the minimum Redundancy Maximum Relevance (mRMR) algorithm was applied for supervised feature ranking. The top 80 most informative features were retained for subsequent modeling, achieving a balance between discriminative power and computational efficiency while mitigating the risk of overfitting. For classification and comparative analysis, three representative classifiers were implemented: (i) Support Vector Machine (SVM): A multiclass SVM with a radial basis function (RBF) kernel was employed. The Error-Correcting Output Codes framework was used to address the four-class classification problem. The kernel scale was automatically determined (“auto”), eliminating the need for manual feature scaling. (ii) Random Forest (RF): A bagging-based ensemble model consisting of 200 decision trees was constructed. The maximum number of splits per tree was limited to 20 to control model complexity and reduce overfitting. Owing to its robustness to noise and feature redundancy, the RF classifier is well suited for medium-scale EEG datasets. (iii) Deep Neural Network (DNN): A fully connected neural network with two hidden layers was designed, comprising 256 and 128 neurons, respectively. ReLU activation functions were applied, followed by batch normalization and dropout (p = 0.5) after each hidden layer for regularization. A Softmax output layer and categorical cross-entropy loss were employed for multiclass classification. To rigorously assess the proposed framework under realistic cross-subject conditions, a five-fold subject-independent cross-validation scheme was adopted. In each fold, training and testing sets were composed of mutually exclusive subjects, ensuring that performance evaluation reflected the model’s generalization capability to unseen individuals.

## Experiments

### Dominant emotion marker

This study is based on the public DEAP EEG emotion dataset and utilizes the accompanying subjective emotion rating files. After viewing each video, participants were asked to rate their emotional responses along four dimensions—Valence, Arousal, Dominance, and Liking (VADL)–which serve as the foundational information for constructing video-level dominant emotion labels. Given the well-recognized variability in emotional experience across individuals, we propose a dynamic hierarchical label calibration strategy aimed at providing a more robust and expressive framework for video-dominant emotion labeling. The primary goal of this strategy is to enhance label stability and improve the representational fidelity of emotion categorization at the group level. Following the widely adopted Valence-Arousal emotion model^45^, the emotional space is divided into four quadrants, each mapped to a representative discrete emotion to facilitate interpretation in subsequent experiments: HVHA (high valence, high arousal) corresponds to happy, HVLA (high valence, low arousal) to relaxed, LVHA (low valence, high arousal) to fearful, and LVLA (low valence, low arousal) to sad.

As an initial baseline, the conventional threshold-based division method was applied to categorize subjects’ emotional responses into the four Valence–Arousal quadrants. This approach achieved an integration accuracy of 92.5% at the individual score level. However, although a label consistency of 50% was observed under individual-based partitioning, the method demonstrated limited effectiveness in modeling dominant emotions at the group level. A statistical decision-making strategy incorporating group voting was further employed to determine video-level dominant emotions, yet the resulting video consistency on the test set reached only 54%. Moreover, the derived labels exhibited a notable deviation from the reference emotion annotations provided in the DEAP dataset. To improve both expression stability and group-level consistency—and to overcome the high subjectivity and weak generalization associated with fixed-threshold or rule-based segmentation methods–we compared three clustering-based labeling strategies. Among them, the Gaussian Mixture Model (GMM) consistently outperformed the alternatives in terms of silhouette coefficients and average consistency metrics. In particular, GMM achieved higher consistency across the majority of videos, indicating its advantage in capturing shared trends in subjective emotional responses to video stimuli. It should be noted, however, that certain marginal emotional categories exhibit inherently fuzzy boundaries and lack a clearly dominant emotional state, leading to relatively low fitting rates with respect to the original reference scores. This observation suggests that existing clustering approaches remain limited in their ability to reliably characterize neutral or weak emotional states. After applying the proposed hierarchical calibration strategy, all videos could be classified into four dominant emotion categories with improved consistency. Specifically, the method achieved an accuracy of 57.27% under our evaluation mode and demonstrated a closer alignment with the dominant emotion distribution of the DEAP dataset, reaching a fit rate of 77.50%. The detailed comparison results are reported in Table 3.

**Table 3:** Labeling effects of different clustering strategies.

Metric (%)	Clustering Strategy			
	K-Means	GMM	Hierarchical	Proposed
Accuracy	55.08	61.41	49.69	57.27
Fit rate	30.00	37.50	7.50	77.50

**Fig. 2:**
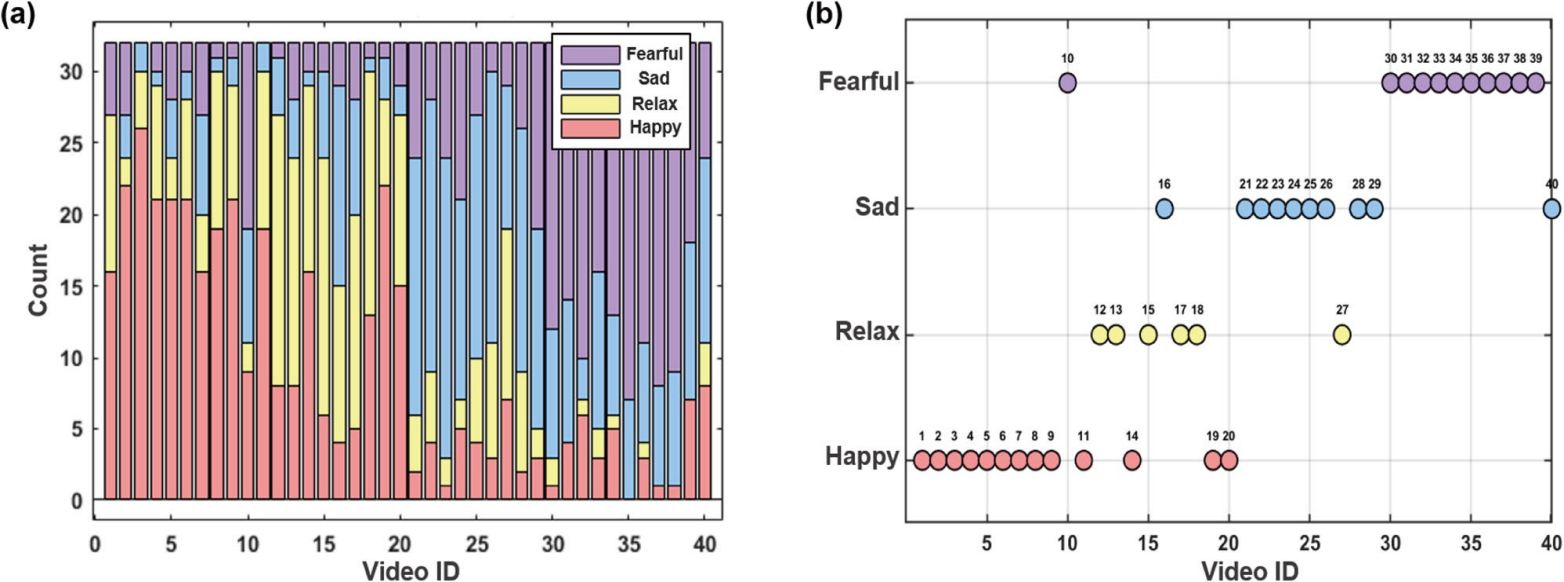
Video sentiment distribution statistics and distribution of dominant emotions across videos (**a**) distribution of participants’ emotional judgments across videos, (**b**) distribution of dominant emotions for each video.

Based on the principle of majority rule in voting theory, the threshold for consistency judgment was set to 50%, ensuring the significance of the identified dominant emotions. In this model, for the 40 videos in the DEAP dataset, the consistency of 9 videos is lower than the set threshold, and the dominant emotion recognition of the rest of the videos has good stability. As shown in Fig. 2 , the distribution of the corresponding four types of emotions are: happy (31.8%), relaxed (19.9%), sad (22.5%), and fearful (25.9%), which can effectively reflect the differences of different videos in guiding people’s emotions, and this innovation significantly improves the ecological validity of the classification.

### Multi-stage signal quality assessment and band-power ratio analysis

Specifically, by analyzing the EEG signals of each subject in the $\delta$ (1–4 Hz), $\theta$ (4–8 Hz), $\alpha$ (8–14 Hz), $\beta$ (14–30 Hz) and $\gamma$ (30–60 Hz) frequency bands of the FFT and Wavelet energy shares were counted to reveal the energy concentration trends of different channels in each frequency band. Based on this, a multi-dimensional energy ratio analysis model was designed.

**Table 4:** EEG channel frequency band power distribution across emotional states (units: %).

Conditions	States	FP1	FP2	F3	F4	C3	C4	T7	T8	PZ	O1	O2
$\delta$ (FFT)	HVHA	**50.06**	35.54	**43.27**	**33.55**	29.97	27.79	29.43	**39.63**	**32.36**	30.04	35.54
	LVHA	27.76	31.42	33.69	27.02	21.30	**14.86**	28.66	34.33	26.22	26.12	31.42
	LVLA	24.10	**40.53**	32.22	27.40	21.34	25.66	26.23	24.52	26.88	**18.33**	**40.53**
	HVLA	31.03	36.44	31.00	27.36	33.12	20.38	23.03	24.61	23.93	27.87	36.44
$\delta$ (Wavelet)	HVHA	**27.52**	23.74	**27.20**	18.34	21.45	14.64	18.91	24.17	20.63	15.12	23.74
	LVHA	15.65	**16.64**	18.72	18.44	9.98	**5.57**	15.74	19.76	12.34	14.23	16.64
	LVLA	15.96	25.08	20.08	17.02	10.66	16.06	17.68	14.45	16.94	11.65	25.08
	HVLA	18.14	23.30	15.38	17.99	19.61	12.79	14.08	12.38	11.42	16.42	23.30
$\theta$ (FFT)	HVHA	17.34	16.39	16.92	16.30	17.84	19.55	15.13	15.57	19.35	16.50	16.39
	LVHA	20.70	18.59	19.00	20.79	24.42	**24.88**	**21.59**	16.22	21.43	21.53	18.59
	LVLA	16.03	19.50	17.60	18.00	28.16	15.00	17.46	15.25	15.31	17.17	19.50
	HVLA	20.19	18.80	21.56	17.11	19.60	16.38	17.53	17.84	22.99	22.83	18.80
$\theta$ (Wavelet)	HVHA	**25.27**	15.04	19.18	18.10	12.20	14.61	13.20	18.76	16.38	16.32	15.04
	LVHA	16.01	16.06	16.44	14.53	12.88	10.18	15.57	17.16	16.98	16.01	16.06
	LVLA	12.22	18.44	16.68	13.56	12.14	12.15	14.64	14.16	13.30	10.30	18.44
	HVLA	19.27	18.67	18.88	14.04	**18.28**	11.52	12.32	15.86	14.43	15.33	18.67
$\alpha$ (FFT)	HVHA	10.78	15.96	20.90	25.68	**16.02**	26.05	**17.07**	22.94	21.74	18.25	15.96
	LVHA	23.55	24.83	23.62	26.84	26.11	29.37	24.15	30.09	21.46	23.83	24.83
	LVLA	**27.35**	20.88	**26.14**	27.52	32.69	31.91	28.21	**37.32**	**27.66**	20.64	20.88
	HVLA	22.43	23.27	21.97	26.73	22.26	26.14	23.34	21.71	22.17	21.81	23.27
$\alpha$ (Wavelet)	HVHA	16.24	14.18	16.83	16.82	17.39	19.79	14.42	15.39	17.73	16.54	14.18
	LVHA	**19.87**	19.90	19.89	18.75	23.96	**25.00**	**21.20**	18.82	20.45	20.10	19.90
	LVLA	16.05	19.84	16.50	17.20	**31.00**	14.20	16.51	16.70	16.39	17.40	19.84
	HVLA	16.66	16.09	18.71	17.30	17.85	16.60	17.83	14.98	21.81	22.83	16.09
$\beta$ (FFT)	HVHA	16.15	**28.05**	13.73	18.17	23.49	22.38	33.95	16.55	22.34	25.76	**28.05**
	LVHA	20.45	17.33	16.31	20.33	24.64	28.86	19.31	14.45	21.76	21.11	17.33
	LVLA	22.07	13.53	19.29	19.47	14.00	20.87	19.98	17.55	20.64	28.58	13.53
	HVLA	20.28	16.25	19.21	21.58	19.08	28.76	28.52	**27.74**	23.72	20.18	16.25
$\beta$ (Wavelet)	HVHA	**10.46**	18.80	19.32	25.07	16.57	27.46	19.35	21.88	23.92	20.80	18.80
	LVHA	24.00	23.81	22.80	27.01	29.73	34.14	24.98	26.59	23.49	24.82	23.81
	LVLA	26.88	19.68	24.94	27.26	**31.01**	32.41	27.10	**34.52**	27.38	23.17	19.68
	HVLA	22.99	23.70	24.62	25.96	22.76	26.39	25.06	24.74	26.29	22.89	23.70
$\gamma$ (FFT)	HVHA	5.68	4.05	5.18	6.31	12.68	4.23	4.42	5.32	4.22	9.45	4.05
	LVHA	7.54	7.82	7.38	5.01	3.54	2.04	6.29	4.91	9.14	7.41	7.82
	LVLA	**10.45**	5.56	4.75	7.61	3.81	6.57	8.12	5.35	9.51	**15.29**	5.56
	HVLA	6.07	5.25	6.26	7.22	5.94	8.35	7.58	**8.10**	7.19	7.31	5.25
$\gamma$ (Wavelet)	HVHA	20.52	**28.24**	17.48	21.68	**32.39**	23.50	34.13	19.80	21.34	31.22	28.24
	LVHA	24.49	23.60	22.15	21.27	23.45	25.11	22.52	17.66	26.75	24.85	23.60
	LVLA	28.89	16.96	21.80	24.96	15.19	25.17	24.06	20.18	25.99	**37.48**	16.96
	HVLA	22.95	18.24	22.41	24.71	21.50	**32.70**	30.71	**32.04**	26.05	22.53	18.24

Values exhibiting potential feature-related differences are shown in bold.

The results in Table 4 showed that different emotional states exhibited significant feature differences across multiple EEG frequency bands, and the FFT and Wavelet analysis methods showed complementary patterns of dominance. In the $\theta$-band, the HVHA state showed significant prefrontal dominance, and the FFT method detected prominent activation of FP1 and F3 channels, whereas the Wavelet method showed broader left hemisphere involvement. Notably, LVLA states showed enhanced $\theta$ power in the right hemisphere FP2 and O2, and this hemispheric asymmetry was verified in both analysis methods. theta band analysis revealed differences in neural markers of emotional arousal, with the FFT method detecting enhanced theta activity in the central region C4 for HVLA states, while the Wavelet method found a prefrontal FP1 significant response, a difference that may reflect differences in the sensitivity of the analyzed methods to temporal features. $\alpha$ bands showed discriminative features of emotional potency, with the FFT method showing a significant $\alpha$ enhancement of the LVLA state in the right temporal lobe T8, whereas the Wavelet method detected prominent activity in the parietal lobe C3 for the HVLA state, a difference in the distribution of the regions that suggests that $\alpha$ oscillations may be be involved in the processing of different emotional components. High-frequency band analyses revealed that: (i) HVHA states showed right prefrontal FP2 dominance in the $\beta$ FFT band, whereas $\gamma$ Wavelet showed left central region C3 activation. (ii) LVHA states showed a distinct right temporal lobe T8 response pattern in the $\gamma$ band. These findings support a specific association of high-frequency activity with emotional arousal.

Comparison of analytic methods showed that (i) FFT is more sensitive to steady-state features. (ii) Wavelet is superior for transient feature capture. (iii) The $\delta$ and $\alpha$ joint feature works best in emotional potency discrimination. (iv) Combination of $\theta$ and $\gamma$ is superior for emotional arousal discrimination. These findings provide new evidence for a multi-frequency oscillatory theory of emotional neural mechanisms, laying the groundwork for later cross-validation using multi-analytic approaches as well as exploring cross-frequency coupling as a potential marker of emotional integration.

### Multimodal feature extraction and dimensionality reduction

**Fig. 3:**
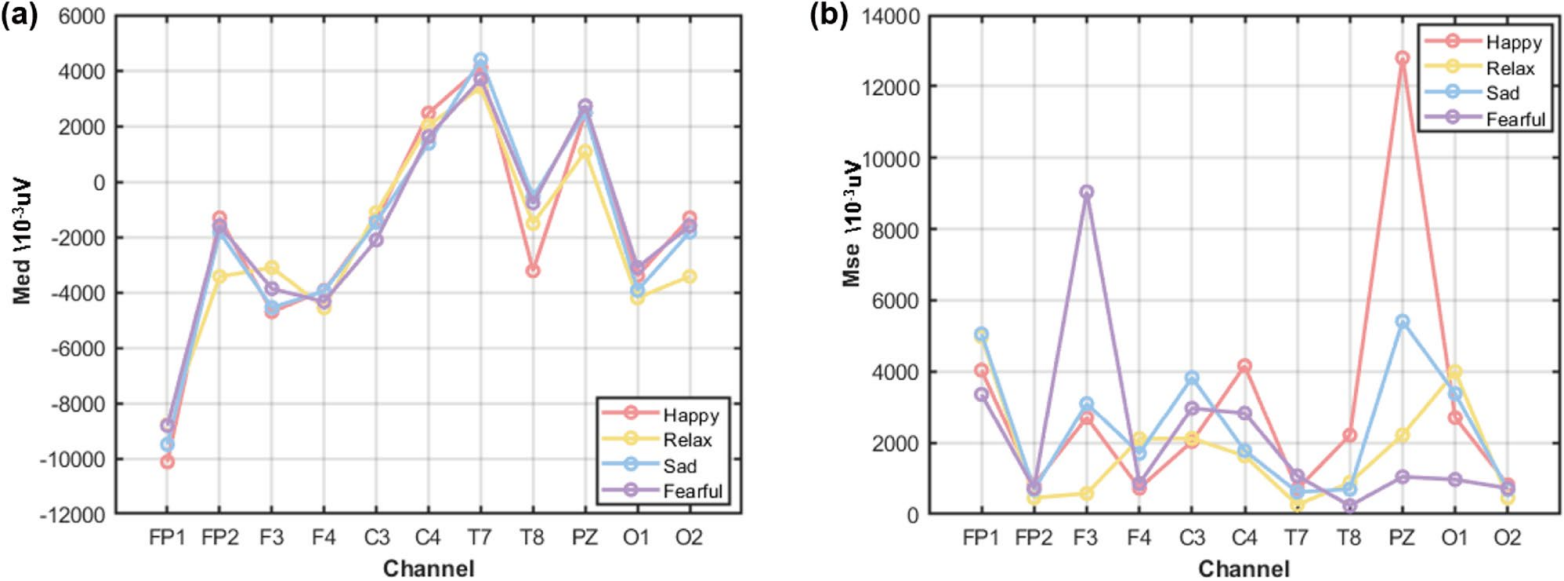
Statistical characteristics of four emotions in different channels (**a**) median of signals, (**b**) Mse of signals.

Figure 3 illustrates the median values and mean square error statistics of EEG signals across different channels under the four emotional conditions. The median serves as a robust estimator of central tendency, reflecting the baseline level of EEG activity while remaining insensitive to outliers. In contrast, the mean square error characterizes the magnitude of signal fluctuations within a given time window; larger values indicate greater dispersion of neural activity, which may correspond to increased cortical activation or neural instability in the associated brain regions. From the channel-wise analysis, several emotion-dependent patterns can be observed. (i) Under the Happy condition, the median amplitudes of the FP1 and T8 channels were notably lower than those observed in other emotional states, suggesting reduced baseline activity in these regions. Meanwhile, the C4 and PZ channels exhibited higher mean square error values, indicating enhanced neural variability and increased activation. (ii) In the Relaxed condition, the median signals of the FP2, PZ, and O2 channels were significantly lower, implying a generally subdued baseline activity in the corresponding cortical areas. In addition, the F3 channel showed a relatively small mean square error, suggesting a more stable or inhibited neural state. (iii) For the Sad condition, the median value of the T7 channel was slightly elevated compared to other emotions, while the C3 channel exhibited increased mean square error, indicating moderate emotional activation in this region. (iv) Under the Fearful condition, the F3 channel displayed a markedly higher MSE, reflecting pronounced neural activation associated with fear processing. In contrast, the FP1, T8, PZ, and O1 channels showed relatively low median values and limited fluctuation amplitudes, suggesting suppressed or less engaged neural activity in these regions.

Motivated by these observations, a saliency-guided feature selection strategy was developed. Multimodal features were extracted by computing the relative energy of each standard frequency band following successive wavelet transforms for four key electrodes (FP1, C3, T8, and O1). For each electrode, energy features from five frequency bands were obtained, yielding a 20-dimensional wavelet energy feature vector. In addition, amplitude-squared coherence was calculated for the F3–C4 electrode pair across five frequency bands, resulting in five coherence-based features. Furthermore, median and peak amplitudes were extracted from band-pass-filtered signals of the emotion-relevant electrodes FP1 and FP2, with particular emphasis on the $\alpha$ and $\beta$ bands, producing a total of 14 time-domain statistical features. Power spectral density (PSD) features were computed using a sliding-window Welch method, including the mean and maximum power in each frequency band across 11 electrodes. This procedure yielded 10 PSD features per electrode, resulting in a 110-dimensional PSD feature set. The final feature vector was constructed by concatenating all extracted features, resulting in a 151-dimensional representation. For cross-subject evaluation, subject identity information was appended to the feature columns. A systematic machine learning pipeline was then employed for model training and evaluation. The dataset consisted of 151-dimensional EEG feature vectors paired with corresponding emotion labels. All raw data were preprocessed using standardized procedures, and the dataset was partitioned into training (70%) and testing (30%) subsets via stratified random sampling. Statistical verification confirmed that the class distributions of the two subsets were well matched ($p > 0.05$). During the feature selection stage, three complementary evaluation methods–mRMR, chi-square testing, and ReliefF–were jointly applied. Based on the fused ranking results, the eight most discriminative feature dimensions were ultimately selected for classification.

### Classification validation

In the feature construction stage, the original 151-dimensional EEG features were organized into several semantically meaningful modalities, including frequency-band coherence, wavelet energy, time-domain statistics, and PSD. Furthermore, to explore the distinct emotional representations across frequency bands, five sub-feature sets were constructed based on the $\delta$ to $\gamma$ bands. Each sub-set included coherence, wavelet energy, statistical metrics, and corresponding PSD features. All feature subsets were independently z-score normalized to ensure numerical stability during model input.

In order to systematically quantify the enhancement effects of each strategy modality on classification performance, a five-fold cross-validation scheme was implemented and ablation experiments were designed to compare the accuracy performance of the three widely used models. The final results are summarised in Table 5.

**Table 5:** Comparison of classification accuracy across different strategies and models (units: %).

Models	Classification strategies		
	Dynamic hierarchical label calibration	Multi-dimensional energy ratio analysis	Saliency-guided feature selection
SVM	34.65 ± 3.5	42.75 ± 3.8	43.98 ± 5.5
RF	32.48 ± 7.4	40.46 ± 4.2	45.82 ± 4.4
DNN	33.08 ± 1.4	36.64 ± 4.1	41.23 ± 5.9

The ablation study results demonstrate the incremental contributions of the three proposed strategies. The baseline performance using Dynamic Hierarchical Label Calibration shows moderate accuracy across all models, with SVM achieving 34.65%, RF at 32.48%, and DNN reaching 33.08%. The introduction of Multi-dimensional Energy Ratio Analysis consistently improved performance for all classifiers, particularly benefiting SVM which increased to 42.75%. The most significant improvement was observed with the Saliency-guided Feature Selection strategy, which yielded the highest accuracy values for all three models. SVM achieved 43.98%, RF reached 45.82%, and DNN attained 41.23% accuracy. This represents a clear progression in performance from the baseline through to the most sophisticated strategy. The results indicate that each strategy contributes meaningfully to the overall performance, with the Saliency-guided Feature Selection providing the most substantial enhancement. The consistent pattern of improvement across all three models suggests that the strategies offer complementary benefits for EEG-based emotion recognition. The standard deviation values further indicate that the performance gains are statistically consistent across cross-validation folds. These findings confirm that each component of the proposed framework contributes incrementally to the final performance, validating the design choices made in this study.

**Table 6:** Performance comparison of three models across evaluation metrics.

Accuracy (%) across 5-fold cross-validation				Performance metrics by emotion category			
Fold	SVM	RF	DNN	Category	SVM	RF	DNN
Fold 1	53.25	48.05	46.75	Recall			
				HVHA	0.629	0.644	0.538
				HVLA	0.030	0.000	0.091
Fold 2	43.59	51.28	44.87	LVHA	0.320	0.402	0.361
				LVLA	0.535	0.535	0.473
				F1-score			
Fold 3	38.71	41.94	34.41	HVHA	0.517	0.535	0.486
				HVLA	0.057	0.000	0.123
				LVHA	0.376	0.459	0.383
Fold 4	39.13	40.22	33.70	LVLA	0.473	0.471	0.444
				Precision			
				HVHA	0.439	0.457	0.444
Fold 5	45.24	47.62	46.43	HVLA	0.500	0.000	0.188
				LVHA	0.456	0.534	0.407
				LVLA	0.423	0.421	0.418

Based on the Table 6, the experimental results demonstrate several positive findings in EEG-based emotion recognition. The proposed method achieves stable performance in detecting HVHA states across all models, with F1-scores maintained at 0.486 for DNN, 0.517 for SVM, and 0.535 for Random Forest. This indicates consistent capability in recognizing positive high-arousal emotions, which is valuable for applications requiring detection of engaged or excited states. The RF model shows the best performance in HVHA emotion recognition, achieving an F1-score of 0.535. This suggests its suitability for capturing the neural signatures of positive engagement. Additionally, all models demonstrate consistent performance in identifying LVLA states, with F1-scores ranging from 0.444 to 0.473, indicating reliable detection of calm or neutral emotional states. The results also identify potential directions for optimization. While HVLA emotions present challenges, the DNN model shows some capability in this category with an F1-score of 0.123, suggesting potential for improvement through targeted feature engineering. The generally balanced performance across valence-arousal dimensions indicates that the feature selection strategy captures the multidimensional nature of emotions. The consistent results across multiple evaluation metrics and cross-validation folds provide evidence for the robustness of the approach. These findings support the feasibility of achieving reliable emotion recognition using optimized electrode configurations, which could benefit practical applications in real-world settings where hardware simplicity is important.

## Discussion

### Comparison with existing DEAP-based studies

Most existing studies using the DEAP dataset aim to improve emotion classification performance at the trial level or within short EEG time windows. These approaches typically rely on high-density EEG configurations (32 channels) and subject-dependent or mixed training strategies, under which high classification accuracy can be achieved. In contrast, the present study is designed around a fundamentally different objective. Rather than optimizing absolute classification accuracy, it focuses on the stability and discriminability of video-induced dominant emotional tendencies at the group level. To this end, more challenging experimental conditions are considered, including video-level emotion labeling instead of instantaneous annotations derived from short EEG segments. Under such settings, direct numerical comparison with DEAP-based studies conducted under high-density, subject-dependent paradigms is not strictly appropriate, as the underlying research goals and evaluation protocols differ substantially. Previous studies have consistently reported pronounced performance degradation when cross-subject constraints and channel reduction are imposed on the DEAP dataset. The performance trends observed in this work are in line with these reports, underscoring the inherent difficulty of EEG-based emotion recognition under conditions that more closely approximate real-world application scenarios. Table 7 provides a methodological comparison of representative EEG-based emotion recognition studies, highlighting key differences in evaluation paradigms, emotion targets, and research objectives between prior work and the present study.

**Table 7:** Methodological positioning of recent EEG-based emotion recognition studies.

Study	Evaluation	Emotion target	Primary goal
Zhang et al.^4^	Subject-dependent	Instantaneous emotion	Maximize classification accuracy
Cheng et al.^5^	Subject-dependent	Short-term emotion state	Fine-grained feature learning
Kanna et al.^6^	Subject-dependent	Binary / multi-class emotion	Sequence modeling performance
This work	Cross-subject	Dominant emotion tendency	Practical group-level emotion modeling

### Impact of channel reduction and cross-subject setting

The classification performance reported in this study is primarily constrained by the intrinsic challenges of EEG-based emotion recognition. EEG signals are characterized by substantial inter-subject variability, including differences in functional brain organization, spectral distributions, and noise characteristics. Under cross-subject evaluation settings, models are required to learn subject-independent emotional representations from a limited feature space, which is widely acknowledged as a particularly challenging problem. Channel reduction further limits the availability of spatial information. While high-density EEG systems can partially mitigate individual variability through spatial redundancy, reduced-channel configurations inevitably impose a lower theoretical upper bound on achievable performance. Importantly, this performance limitation should not be interpreted as a weakness of reduced-channel strategies. On the contrary, such configurations more accurately reflect the practical constraints of wearable and portable EEG systems in real-world applications. Therefore, although the achieved classification performance may not be numerically optimal, the results offer stronger practical relevance in terms of deployment feasibility and ecological validity.

### Video-level emotion modeling versus instantaneous classification

Emotion elicited by video stimuli is not equivalent to the emotional state reflected by instantaneous EEG segments. In the DEAP dataset, emotional labels are derived from subjective ratings of entire video clips, representing time-integrated dominant emotional tendencies rather than momentary emotional fluctuations. Directly predicting video-level labels from short EEG segments therefore introduces a temporal mismatch between signal representation and annotation targets. To address this issue, the present study employs consistency analysis and clustering to characterize video-induced dominant emotional patterns at the group level, rather than assigning potentially unstable labels to individual EEG segments. This perspective aligns more closely with the formation mechanism of emotional experience in video-based paradigms and helps reduce randomness caused by transient noise and individual variability. Accordingly, the focus of this study is not extreme accuracy in instantaneous emotion classification, but the feasibility of reliably capturing and distinguishing dominant emotional tendencies at the EEG level.

### Methodological dependency and future directions

Although the proposed method demonstrates a degree of robustness under reduced-channel and cross-subject conditions, its applicability boundaries should be acknowledged. The method relies on the experimental paradigm specific to the DEAP dataset, including video-based stimulation, valence–arousal rating dimensions, and the associated labeling procedure. Direct transfer to datasets with different stimulus modalities or labeling schemes may therefore require adjustments in label construction and feature aggregation. Moreover, this study emphasizes within-paradigm analysis rather than cross-dataset generalization, and the method has not been systematically evaluated on heterogeneous emotion datasets. This constitutes a primary limitation of the present work and a clear direction for future research. Future studies may incorporate additional datasets with similar experimental designs to further assess the generalizability of video-dominant emotion modeling under reduced-channel and cross-subject constraints. In addition, integrating the proposed framework with advanced temporal modeling or self-supervised learning approaches may offer further improvements in group-level emotional consistency analysis.

## Conclusions

This paper investigates video-based emotion recognition using portable EEG devices, a setting in which reduced electrode configurations and cross-subject variability pose significant challenges to conventional approaches. Instead of focusing exclusively on maximizing classification accuracy, this study emphasizes the modeling of video-level dominant emotional tendencies, which more closely aligns subjective annotations with sustained neural responses elicited during video viewing. From a methodological perspective, the proposed hierarchical label calibration strategy elucidates how individual consistency and boundary ambiguity jointly affect the reliability of emotion labels in EEG-based affective analysis. When combined with time–frequency energy ratio features and saliency-guided feature selection, the framework offers insight into how emotionally relevant information can be effectively preserved under constraints of limited channel availability and subject-independent evaluation. From a practical standpoint, the experimental results support the feasibility of reduced-channel EEG emotion recognition for portable and wearable applications, where sensor simplicity and computational efficiency are critical considerations. The proposed framework provides a structured approach to balancing recognition robustness and lightweight system design, making it particularly relevant for real-world affective monitoring scenarios. Several limitations remain. First, the current evaluation is conducted on a single dataset, and the robustness of the proposed strategy across diverse populations, recording configurations, and emotion elicitation protocols has yet to be established. Second, although the feature extraction pipeline is designed with efficiency in mind, further simplification will be required to fully satisfy the real-time processing constraints of embedded and edge-computing platforms.

Future work will therefore focus on cross-dataset validation and on the development of low-latency feature representations that better support online or edge-based emotion recognition. In particular, the video-based stimulation protocol and structured subjective rating scheme of the DEAP dataset provide a useful reference for designing future data collection procedures under similar application constraints. Extending this experimental paradigm may facilitate more consistent label construction and enable systematic evaluation of video-dominant emotion modeling in newly collected datasets. Overall, this work represents a practically grounded and conceptually coherent step toward cross-subject EEG-based emotion analysis in portable and resource-constrained settings.

## Data Availability

The data analyzed in this study were obtained from the publicly available DEAP dataset, accessible at http://www.eecs.qmul.ac.uk/mmv/datasets/deap/.

## Code availability

Code and models used in this study are publicly available at Zenodo: https://doi.org/10.5281/zenodo.18420002.
